# Inoculation with *Lentilactobacillus buchneri* alone or in combination with *Lentilactobacillus hilgardii* modifies gene expression, fermentation profile, and starch digestibility in high-moisture corn

**DOI:** 10.3389/fmicb.2023.1253588

**Published:** 2023-10-12

**Authors:** Pascal Drouin, Érica Benjamim da Silva, Julien Tremblay, Eric Chevaux, Emmanuelle Apper, Mathieu Castex

**Affiliations:** ^1^Independent Researcher, Saint-Jean-sur-Richelieu, QC, Canada; ^2^Lallemand Brasil LTDA, Aparecida de Goiânia, Brazil; ^3^Energy, Mining, and Environment, National Research Council of Canada, Montréal, QC, Canada; ^4^Lallemand SAS, Blagnac, France

**Keywords:** gene expression, high-moisture corn, inoculant, *Lentilactobacillus buchneri*, *Lentilactobacillus hilgardii*, silage, metatranscriptomics

## Abstract

Inoculants combining *Lentilactobacillus buchneri* and *Lentilactobacillus hilgardii* have been shown to improve the aerobic stability of high-moisture corn (HMC) and whole-plant corn silage, but the mode of action of this co-inoculation remains to be elucidated. This study used metatranscriptomics to evaluate the effects of inoculation with *L. buchneri* alone or combined with *L. hilgardii* on the bacterial community, gene expression, fermentation profile, and starch digestibility in HMC. High-moisture corn not inoculated (Control) or inoculated with *L. buchneri* NCIMB 40788 (LB) or *L. buchneri* NCIMB 40788 combined with *L. hilgardii* CNCM-I-4785 (Combo) was ensiled in mini silo bags for 30, 60, 120, and 180 days. The fermentation profile was evaluated at all time points. Metatranscriptomics was performed on samples collected on day 120. Combo had a greater alpha diversity richness index of contigs than LB and Control, and inoculation with Combo and LB modified the beta-diversity of contigs compared to Control. Out of 69 genes of interest, 20 were differentially expressed in LB compared to Control and 25 in Combo compared to Control. Of those differently expressed genes, 16 (10 of which were associated with carbohydrate metabolism and six with amino acid metabolism) were differently expressed in both LB and Combo compared to Control, and all those genes were upregulated in the inoculated silages. When we compared Combo and LB, we found seven genes expressed differently, four associated with carbohydrate metabolism and downregulated in Combo, and three associated with amino acid metabolism and upregulated in Combo. At day 120, the inoculated silages had more culturable lactic acid bacteria, higher *Lactobacillus* relative abundance, and lower *Leuconostoc* relative abundance than Control. The concentration of acetic acid remained low throughout ensiling in Control, but in LB and Combo, it increased up to day 60 and remained stable from day 60 to 180. The 1,2-propanediol was only detected in LB and Combo. Inoculation did not affect the concentration of starch, but starch digestibility was greater in Combo than in Control. Inoculation of HMC with Combo modified the gene expression and fermentation profile compared to Control and LB, improving starch digestibility compared to uninoculated HMC.

## 1. Introduction

High-moisture corn (HMC) is a high-energy silage mainly fed to dairy cattle. Compared to dry corn grain, HMC has the advantage of greater starch digestibility (starchD) but the drawback of being more prone to microbial activity due to its higher moisture content and concentration of available sugars (Kung Jr et al., 2007; Ferraretto et al., 2013). Because of its susceptibility to microbial activity, HMC is at high risk of aerobic deterioration, initiated by yeasts that assimilate lactic acid and increase silage pH (Pahlow et al., 2003). It is recognized that ensiling increases starch degradability in the rumen thanks to the proteolysis of the hydrophobic zein proteins in the starch-protein matrix (Hoffman et al., 2011). Therefore, improving starch digestibility while simultaneously controlling aerobic deterioration is a challenge for HMC production. Fortunately, some tools can help circumvent those challenges, such as inoculants based on lactic acid bacteria (LAB).

The digestibility of HMC can be improved by increasing the degradation of the starch-protein matrix of the corn kernels either physically or chemically. For example, grinding the corn kernels can increase total tract starchD (Saylor et al., 2021). Chemical degradation of the starch-protein matrix in silage occurs through proteolysis driven mostly by bacterial activity (Junges et al., 2017). Even though LAB do not possess high proteolytic activity, inoculation with the obligate heterofermentative LAB *Lentilactobacillus buchneri* has been associated with increased proteolysis and improved starch digestibility (da Silva et al., 2019; Saylor et al., 2020). It has been suggested that *L. buchneri* facilitates proteolytic bacteria activity by reducing environment acidity by converting the stronger acid (lactic acid), into the weaker acid, (acetic acid; Oude Elferink et al., 2001; Junges et al., 2017). Even though inoculation with *L. buchneri* could improve starch digestibility, inoculants containing obligate heterofermentative LAB, such as *L. buchneri*, *Lentilactobacillus hilgardii*, *Lentilactobacillus brevis*, and *Lentilactobacillus diolivorans*, are mainly used in silage making to produce acetic acid (Muck et al., 2018). Acetic acid has antifungal properties and can control the development of yeasts that cause aerobic deterioration (Moon, 1983; Kung Jr et al., 2018). Combining different LAB species in a single inoculant formulation is widely used to improve efficacy, reduce costs, broaden the action spectrum, and reduce silage curing time. For example, inoculating HMC with a combination of *L. buchneri* and *L. hilgardii* has been shown to increase the production of antifungal compounds and to greatly improve aerobic stability after 10 d of ensiling compared to non-inoculated silage and silage inoculated with *L. buchneri* or *L. hilgardii* separately (da Silva et al., 2021).

Although some studies have shown the benefits of co-inoculating *L. buchneri* and *L. hilgardii*, the mode of action of this combination on ensiling is still undetermined (Drouin et al., 2019; Ferrero et al., 2019; da Silva et al., 2021). McAllister et al. (2018) suggested that metatranscriptomics could be a useful tool to investigate the expression of genes related to carbohydrate degradation and proteolysis, as this technology could shed light on the mode of action of *L. buchneri* and *L. hilgardii* co-inoculation. A few published studies have used *in silico* tools to predict changes in microbial function based on metagenomic data on silage in response to inoculation (Bai et al., 2021; Xu et al., 2021). However, to the best of our knowledge, no studies have been published that used metatranscriptomics to investigate the effects of inoculants on gene expression in silage. Therefore, this work aimed to investigate the effects of inoculation with *L. buchneri* alone or combined with *L. hilgardii* on the bacterial community, transcriptome, fermentation profile, and starch digestibility in HMC.

## 2. Materials and methods

### 2.1. Ensiling and experimental treatments

The corn kernels were gathered from Rover’s Farm Inc. (Chazy, NY, United States) on October 25th by a T670 corn combine harvester (John Deere, Moline, IL, United States) and stored at outdoor temperature for 18 h before milling. The kernels were then thoroughly mixed to ensure homogeneity and milled using meat grinders. The mesh size was 8 mm and the size distribution of the milled kernels ranged from 0.04 to 5.34 mm (median 1.08 mm), a distribution pattern in line with that in published data (Bitra et al., 2008; Wongsagonsup and Jane, 2017) for hammer milling. The mean dry matter (DM) content of the milled kernels was 708.9 ± 5.2 g per kg.

The milled kernels were thoroughly mixed and divided into 15 piles each weighing 2.50 kg (three treatments × five repetitions) prior to application of the treatments. The three treatments consisted of water (Control), *L. buchneri* NCIMB 40788 at 400,000 CFU g^−1^ of fresh forage (LB), and a combination of two *Lentilactobacillus* strains (*L. buchneri* NCIMB 40788 and *L. hilgardii* CNCM-I-4785; Combo) each applied at 200,000 CFU g^−1^ of fresh forage. The treatments were prepared in a total volume of 300 mL to ensure the homogeneity of the cell suspension and 50 mL was applied to each pile by alternating spraying and mixing, corresponding to a 2% increase in the moisture content. After treatment, each pile was thoroughly mixed and then four 300 g fractions (one per storage period) of the treated milled kernels were transferred to a plastic bag (20 × 30 cm, 2-ply 3 mil polyethylene, Western Brands LLC, Ohio), and vacuum sealed using a commercial vacuum sealer (Model 3,000, Weston Brands LLC, Ohio). A 200 g sample from each pile was frozen for chemical analysis. The treatments were applied sequentially, starting with the Control, then LB, and finally Combo, to avoid contamination. The mini-silos were stored at ambient temperature (~21°C) for 30, 60, 120, and 180 days of fermentation. A total of 60 mini-silos were prepared according to a completely randomized experimental design consisting of three inoculation treatments, four storage periods, and five replicates.

### 2.2. Sampling, fermentation profile, and starch digestibility

At each opening, except for the samples taken on day 120 (see following RNA isolation section), one set of mini-silo bags was collected and immediately frozen at −20°C. These samples were sent to Cumberland Valley Analytical Services (Pennsylvania, United States) for chemical analysis and quantification of the pH and the concentrations of lactic acid, acetic acid, 1,2-propanediol, ethanol, total volatile fatty acids (VFA), and NH_3_-N. Starch disappearance was quantified in all samples using the 7-h ruminal *in vitro* starch digestibility protocol developed by Cumberland Valley Analytical Services (Pennsylvania, United States) on aliquots of the samples dried and ground using a knife mill and a 4 mm sieve based on the method described by Goering and Van Soest (1970). Dry matter was adjusted for volatiles according to the equation of Dahlke and Goeser (2022).

### 2.3. RNA isolation and microbial enumeration following the 120-day silo opening

After 120 days of fermentation, the mini-silos were opened by cutting the bags open using a sterile scalpel, and 1 g of the silage was collected from the center of the bag in a centrifuge tube. Liquid nitrogen was added, and the tubes were immediately stored at −80°C for RNA extraction. A 10-g sample was also collected and stored at 4°C for microbial enumeration on agar plates. After collecting those samples, the bags with the residual HMC silage were placed in new bags, sealed under a low vacuum, and stored at −20°C for chemical analysis. RNA samples that were stored at −80°C were subjected to extraction using the Quick-RNA Miniprep Plus kit (Zymo Research, Irvine, California, United States) immediately after being removed from the freezer. For cell counts, the 10-g samples were suspended in 90 mL of a peptone-NaCl buffer and mixed for two 1-minute cycles using a Stomacher 400 paddle blender (Seaward, United Kingdom), followed by serial dilutions with a peptone-NaCl buffer. Lactic acid bacteria were enumerated on de Man Rogosa Sharpe agar (Oxoid, UK) containing 100 μg/L of cycloheximide (Goering and Van Soest, 1970; Drouin and Ferrero, 2020). All the plates were incubated at 28°C and cell counts were performed after 72 h.

For each sample, a 200 mg aliquot was added to the ZR BashingBead Lysis Tube from the Quick-DNA fecal/soil microbe miniprep kit (Zymo Research, Irvine, CA, United States) along with 750 μL of Bashing buffer (Quick-DNA Fecal/Soil Microbe Miniprep Kit, Zymo Research) treated by adding 1% DEPC. The samples were placed in a Mixer Mill (Retsch, Germany) for bead beating for 120 s at 25 rpm and centrifuged for 60 s at 10000 g at 4°C. The samples were kept on ice in all the extraction steps except for the bead beating step, and all the centrifugation steps were performed at 4°C. Following centrifugation, an aliquot of 400 μL was used for the following step of the RNA purification protocol. The DNase I option of the protocol was selected (step 3), consisting of the recommended DNA digestion treatment using 5 μL of DNase I (1 U/μl) to 75 μL of DNA digestion buffer and followed by incubation at room temperature for 15 min. RNA samples were stored at −80°C until they were sequenced at the National Research Council of Canada, Montreal, Canada.

### 2.4. Library preparation and sequencing

RNA samples were quantified using the Quant-iT^™^ RiboGreen^™^ RNA Assay Kit (Invitrogen Cat No R11490) according to the manufacturer’s protocol for a 200 μL assay, which was slightly modified by reducing the final volume to 100 μL. The fluorescence readings were performed with a Tecan Infinite^®^ M1000 Microplate Reader (Tecan, Männedorf, Switzerland). The first three of the five replicates from each treatment were selected for RNA sequencing.

PCR amplifications were performed with 16S primers to confirm the absence of DNA in each sample. The presence of faint 16S product bands prompted us to repeat the DNase treatment using the TURBO DNA-free^™^ Kit (Invitrogen Cat No AM1907) according to the manufacturer’s protocol with the modification that two 30-min rounds of DNase treatment at 37°C were performed back-to-back with more DNase added before the second incubation at 37°C. After purification, 16S PCR amplifications confirmed the absence of DNA in each sample.

DNA-free RNA samples were quantified to prepare libraries using the Quant-iT^™^ RiboGreen^™^ RNA Assay Kit. A quantity ranging from 50 ng to 700 ng was used to prepare the library using the ScriptSeq^™^ Complete Kit (Bacteria)–Low Input (Illumina Cat No BB1224) according to the manufacturer’s protocol. Briefly, the RNA samples were depleted for ribosomal RNA (rRNA) using the included Ribo-Zero kit. After rRNA depletion, the RNA was purified using RNAClean XP (Agencourt Cat No A63987 from Beckman-Coulter) according to the manufacturer’s protocol. The rRNA-depleted RNA was then fragmented and transcribed into double-stranded cDNA (ds-cDNA). The ds-cDNA was end-repaired and double-tagged at each end with Illumina adapters. After each of these steps, the DNA was purified using AMPure XP (Agencourt Cat No A63880) according to the manufacturer’s protocol. Final PCR amplification was performed to add indexes (ScriptSeq Index PCR Primers Set 1, Illumina Cat No RSBC10948) to each sample to allow multiplexing of the samples during sequencing on an Iseq PE 100 bp run. After indexing, each library was evaluated and quantified with a bio-analyzer 4,200 TapeStation System (Agilent Technologies) using the High Sensitivity D5000 ScreenTape system and reagents (Agilent Cat Nos 5,067–5,592 and 5,067–5,593). The pool was repurified on SPRIselect beads (Agencourt Cat No B23318) to remove adapter dimers. The pool was then sequenced on two Iseq (Illumina) lanes using the Paired-End 100 Base Pair Kit.

### 2.5. Bioinformatics

Nine samples (three replicates per treatment) were submitted for sequencing, and the resulting data were processed following the metatranscriptomic bioinformatic pipeline of Tremblay et al. (2017). Sequencing adapters were removed from each read, and bases at the end of reads with a quality score of less than 30 were cut off (Trimmomatic v0.32; Bolger et al., 2014) and scanned for contaminant reads (i.e., sequencing adapters and rRNA reads) using BBDUK^1^ to generate quality controlled (QC) reads. QC-passed reads from each sample were co-assembled using Megahit v1.2.9 (Li et al., 2015) with iterative kmer sizes of 31, 41, 51, 61, 71, 81, and 91 bases. Gene prediction was performed by calling genes on each assembled contig using Prodigal v2.6.2 (Hyatt et al., 2010). QC-passed reads were mapped (BWA mem v0.7.15; unpublished)^2^ against contigs to assess the quality of the metagenome assembly to obtain contig abundance profiles. Alignment files in BAM format were sorted by read coordinates using SAMtools v1.2 (Li et al., 2009) and only properly aligned read pairs were kept for downstream steps. Each BAM file (containing properly aligned paired-reads only) was analyzed for coverage of called genes and contigs using bedtools (v2.17.0; Quinlan and Hall, 2010) using a bed file representing gene coordinates on each contig. Only paired reads with both overlapping contigs and genes were considered valid counts. Coverage profiles of each sample were merged to generate an abundance matrix (rows = contig, columns = samples) for which a corresponding CPM (counts per million – normalized using the TMM method; edgeR v3.10.2; Robinson et al., 2010). Taxonomic lineages of each contig were assigned using CAT v5.0.3 (von Meijenfeldt et al., 2019). Taxonomic summaries and beta-diversity analyses were performed using MicrobiomeUtils v0.9.4.^3^ Genes were compared against the KEGG genes database (Kanehisa and Goto, 2000; Kanehisa, 2019) using DIAMOND Blastp (v2.0.8; Buchfink et al., 2021). Top matches with at least an e-value <1–10 were assigned their corresponding KEGG ortholog identifier from the KEGG genes database. Alpha diversity richness was computed with RTK v0.93.2 (Saary et al., 2017).

### 2.6. Statistical analysis

Data on alpha diversity, relative abundance (RA), fermentation profile, and starch disappearance were analyzed using the Fit Model procedure of SAS-JMP Pro 16 (SAS Institute Inc.). Alpha diversity and RA data at day 120 were analyzed as a completely randomized design with the effect of inoculation treatment (Control, LB, and Combo). Fermentation profile and starch disappearance data were analyzed as a completely randomized design with a 3-by-5 factorial arrangement of treatments, the main factors being the effect of the inoculation treatment (Control, LB, and Combo), time (0, 30, 60, 120, and 180 d), and their interaction. Tukey’s test (Snedecor and Cochran, 1980) was performed if a main effect or interaction was significant (*p* ≤ 0.05). The RA, fermentation profile, and starch disappearance results were plotted using the ggplot2 package version 3.3.5 (Wickham, 2016) in R version 4.1.2 (R Core Team, 2021).

The following analyses were performed in R version 4.1.2 (R Core Team, 2021). Contrast analysis between LB and Control, Combo and Control, and Combo and LB for gene abundances (CPM relative to the maximum) was performed using the Limma package version 3.50.1 (Ritchie et al., 2015) with the functions lmFit, eBayes, and topTable (Phipson et al., 2016). ANOSIM (analysis of similarity) test was performed using the Vegan package version 2.5–7 (Oksanen et al., 2020) to analyze the significance of the results of principal coordinate analysis (PCoA). The Corrplot package version 0.92 (Wei and Simko, 2021) was used to produce the principal component analysis (PCA) biplot with the relationship between silage samples and chemical variables and Pearson’s correlation matrix between the expression of genes of interest and chemical variables.

## 3. Results

**Figure 1:**
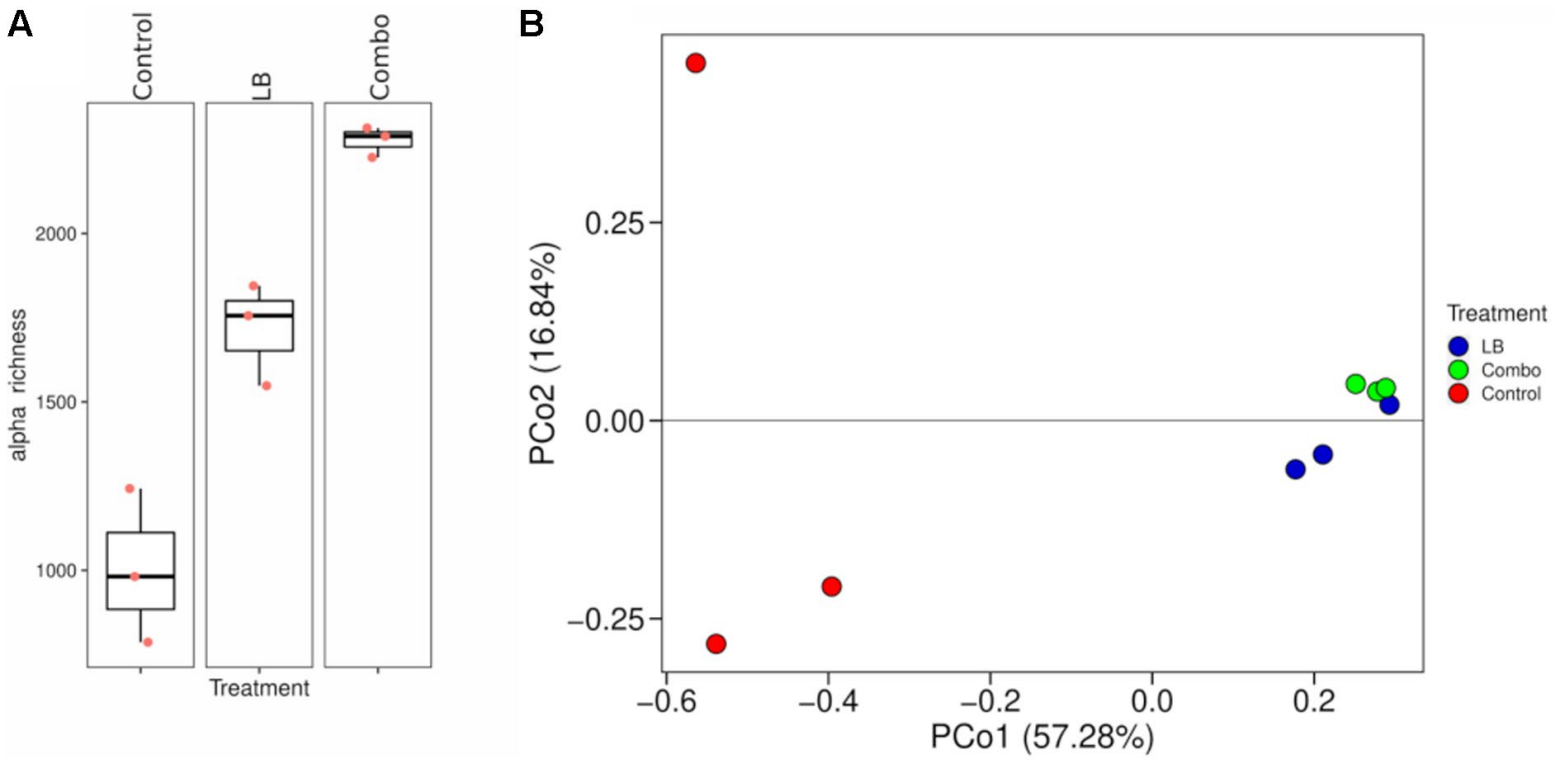
**(A)** Boxplot showing the variation in alpha diversity (richness index) across treatments on rarefied (read depth of 105,432) contig abundance data. The box represents the interquartile range between the first and third quartiles and the line inside the box represents the median of the three replicates (*p* < 0.001, SEM = 94). **(B)** Principal coordinate analysis (PCoA) plot with Bray–Curtis dissimilarity of contig abundance (ANOSIM *p* value = 0.003). Treatments consisted of uninoculated (Control) silage or silage inoculated with *Lentilactobacillus buchneri* NCIMB 40788 at 400,000 CFU/g of fresh weight (LB) or with *L. buchneri* NCIMB 40788 plus *Lentilactobacillus hilgardii* CNCM-I-4785 at 200,000 CFU/g of fresh weigh each (Combo), ensiled for 120 days.

The summary statistics of transcriptome assembly and of the RNA-seq data and mapping results are shown in Tables 1, 2, respectively. The alpha diversity richness index of contigs at a read depth of 105,432 differed among treatments (*p* < 0.05; Figure 1A). The Combo treatment had the highest index (2,275), LB an intermediate index (1,715), and Control had the lowest (1,004) index. The Bray–Curtis dissimilarity analysis had a significant ANOSIM test (*p* = 0.003), indicating a significant difference between at least two out of the three treatments. The PCoA plot shows that LB and Combo clustered close together but separately from Control (Figure 1B).

**Table 1:** Summary statistics of transcriptome assembly.

Statistics	Contigs
Number of contigs (≥ 0 bp)	2,864
Number of contigs (≥ 1,000 bp)	764
Number of contigs (≥ 5,000 bp)	17
Number of contigs (≥ 10,000 bp)	0
Number of contigs	1,508
Largest contig	9,256
Total length (≥ 0 bp)	2,468,267
Total length (≥ 1,000 bp)	1,478,742
Total length (≥ 5,000 bp)	108,173
Total length (≥ 10,000 bp)	0
Total length	2,007,090
GC (%)	45.11
N50	1,581
N75	979
L50	391
L75	791
Number of N’s per 100 kbp	40.36

All statistics are based on contigs of size ≥ 500 bp, unless otherwise noted.

**Table 2:** Summary statistics of RNA-seq data and mapping results.

Sample*	Raw fragments	Percentage of surviving fragments	Surviving single	Total reads QCed	Percentage mapped	Percentage properly paired
Control-1	624,339	75%	12,585	359,588	64%	62%
Control-2	1,708,943	95%	41,450	542,178	72%	70%
Control-3	839,194	59%	16,214	641,254	23%	20%
LB-1	563,448	86%	10,574	269,844	89%	88%
LB-2	1,296,313	92%	25,352	498,284	93%	92%
LB-3	778,411	85%	16,647	775,886	95%	94%
Combo-1	989,241	95%	29,899	1,427,708	75%	73%
Combo-2	1,308,594	64%	29,574	1,361,310	76%	74%
Combo-3	1,169,918	59%	23,062	1,172,700	68%	65%

*Control, uninoculated silage; LB, silage inoculated with *Lentilactobacillus buchneri* NCIMB 40788 at 400,000 CFU/g of fresh weight; Combo, silage inoculated with *L. buchneri* NCIMB 40788 plus *Lentilactobacillus hilgardii* CNCM-I-4785 at 200,000 CFU/g of fresh weight each.

**Figure 2:**
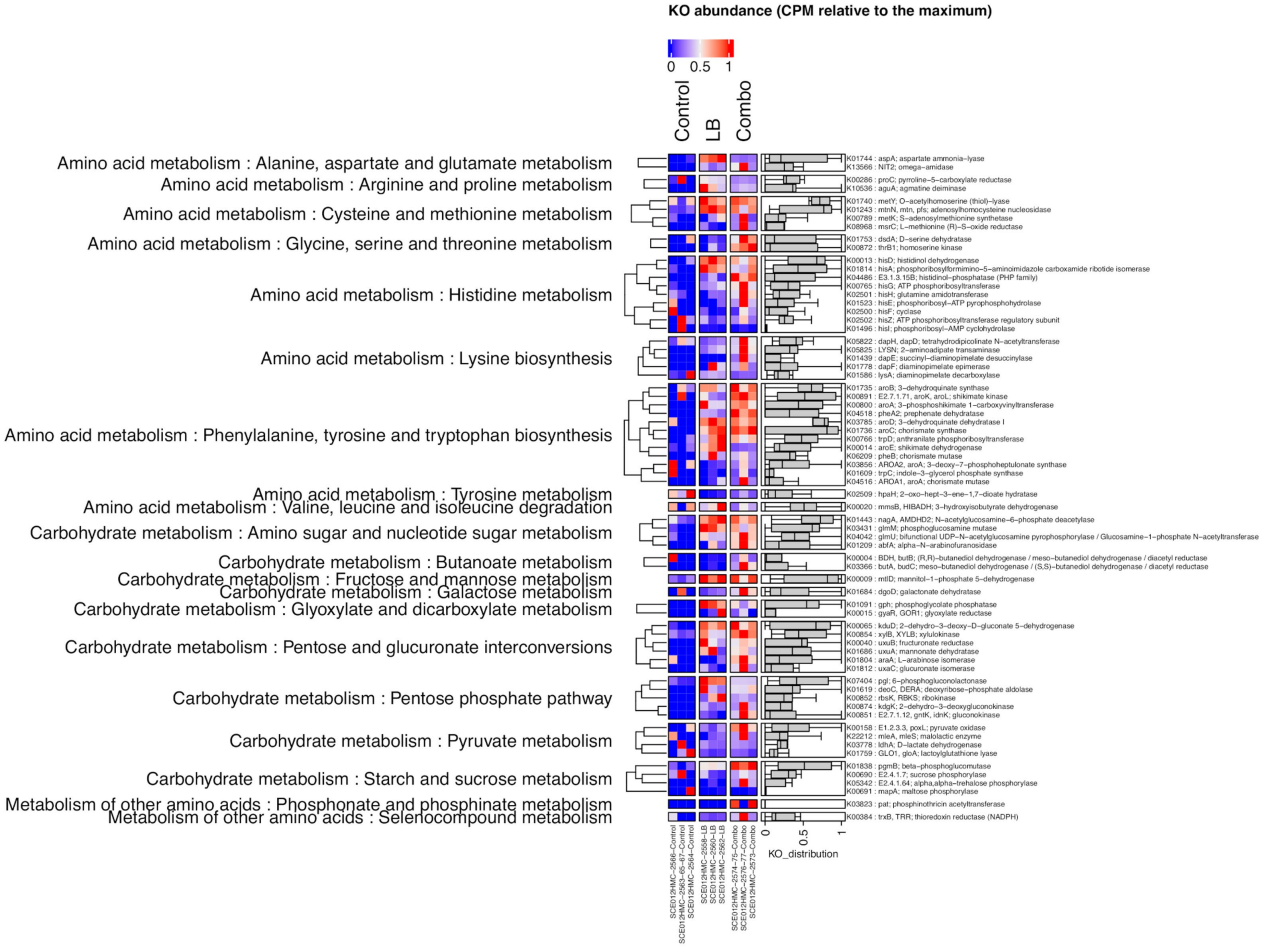
Heatmap of the abundance, expressed as counts per million (CPM) relative to the maximum, of gene (KO) associated with amino acid and carbohydrate metabolism in uninoculated (Control) silage and in silage inoculated with *L. buchneri* NCIMB 40788 at 400,000 CFU/g of fresh weight (LB) or with *L. buchneri* NCIMB 40788 plus *L. hilgardii* CNCM-I-4785 at 200,000 CFU/g of fresh weight each (Combo), ensiled for 120 days.

A heatmap of the abundance of genes (KO) of interest in Control, LB, and Combo silages ensiled for 120 days is shown in Figure 2. Of the 69 genes of interest related to amino acid and carbohydrate metabolism, 20 were differentially expressed between LB and Control and 25 between Combo and Control. Sixteen of these genes were differentially expressed (*P*adj < 0.05) in both LB and Combo compared to Control and were all upregulated in the inoculated silages. Ten of these 16 genes were associated with carbohydrate metabolism, specifically with glyoxylate and dicarboxylate metabolism (*gyaR*), pyruvate metabolism (*poxL*, *ldhA*, and *mleA*/*mleS*), pentose phosphate (PP) pathway (*gntK*/*idnK*, *rbsK*, and *kdgK*), pentose and glucuronate interconversions (*uxaC*), starch and sucrose metabolism (*pgmB*), and amino sugar and nucleotide sugar metabolism (*glmM*). The other six genes were associated with amino acid metabolism, specifically with arginine and proline metabolism (*proC* and *aguA*), lysine biosynthesis (*dapE*), aspartame and glutamate metabolism (*aspA*), glycine, serine, and threonine metabolism (*thrB1*), and seleno-compound metabolism (*trxB*). No gene was downregulated in Combo compared to Control, and only one gene was downregulated in LB compared to Control, *mapA*, a gene associated with starch and sucrose metabolism. Seven genes were differentially expressed (*P*adj < 0.05) in Combo and LB. Four of them were associated with carbohydrate metabolism and were downregulated in Combo compared to LB. Specifically, the genes concerned were associated with pentose and gluconate interconversion (*uxaC* and *uxuB*), with the pentose phosphate pathway (*gntK*/*idnK*), and with glyoxylate and dicarboxylate metabolism (*gyaR*). The other three genes were associated with amino acid metabolism and were upregulated in Combo compared to LB. These genes are involved with alanine, aspartame, glutamate metabolism (*aspA* and *nit2*), and lysine biosynthesis (*dapE*). In addition, the gene coding for the enzyme EC 2.4.1.64 (alpha,alpha-trehalose phosphorylase), associated with starch and sucrose metabolism, tended (*P*adj = 0.07) to be upregulated in Combo compared to LB.

**Figure 3:**
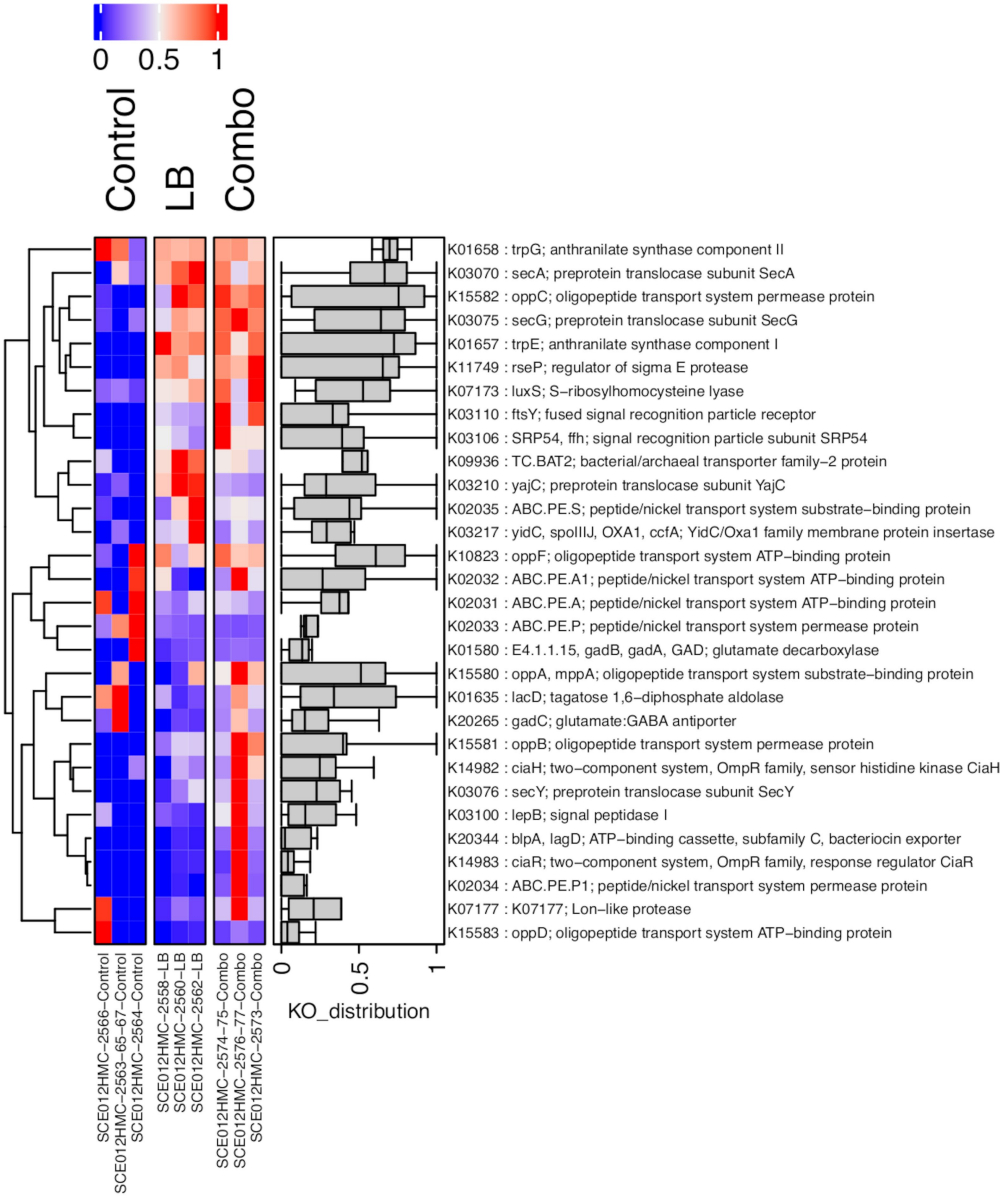
Heatmap of the abundance, expressed as counts per million (CPM) relative to the maximum, of genes (KO) associated with the cellular community (quorum sensing [PATH: KO02024]) in prokaryotes in uninoculated (Control) silage or silage inoculated with *L. buchneri* NCIMB 40788 at 400,000 CFU/g of fresh weight (LB) or with *L. buchneri* NCIMB 40788 plus *L. hilgardii* CNCM-I-4785 at 200,000 CFU/g of fresh weight each (Combo), ensiled for 120 days.

A heatmap with genes associated with quorum sensing is shown in Figure 3. Of the 30 genes identified, 11 were differentially expressed in LB compared to Control, and eight were differentially expressed in Combo compared to Control. Of these genes, six were differentially expressed in both LB and Combo compared to Control. Five of the six genes (*lacD*, *yidC*, *oppD*, genes coding for a Lon-like protease, and genes coding for the protein ABC.PE.S) were upregulated in inoculated silages compared to in Control, and one (*secY*) was downregulated in inoculated silages compared to in Control. The gene *yajC* was the only one differentially expressed in Combo compared to LB, being downregulated in Combo.

**Figure 4:**
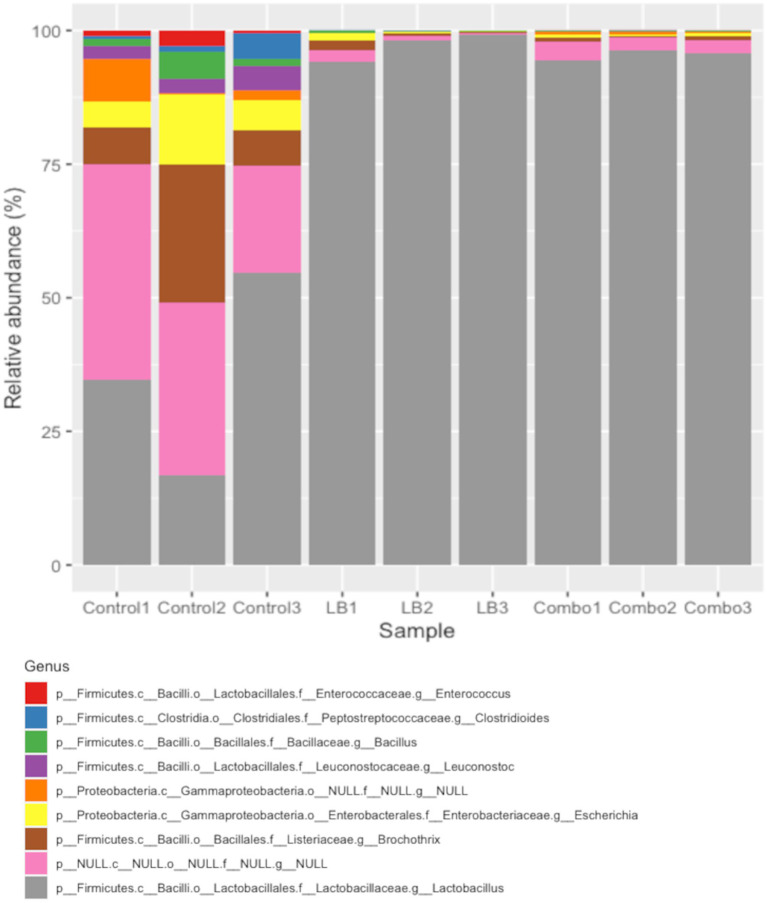
Relative abundance of bacterial genera in silage ensiled for 120 days. Only genera with a relative abundance of more than 0.5% are shown. Treatments consisted of uninoculated (Control) silage or silages inoculated with *L. buchneri* NCIMB 40788 at 400,000 CFU/g of fresh weight (LB) or with *L. buchneri* NCIMB 40788 plus *L. hilgardii* CNCM-I-4785 at 200,000 CFU/g of fresh weight each (Combo).

We also analyzed changes in microbial community composition by inoculation (Figure 4). Approximately 28% of the transcripts associated with the total population of bacteria in Control, 1% in LB, and 2% in Combo, were not identified at the phylum level. *Firmicutes* (83.64%) and *Proteobacteria* (4.84%) were the main phyla identified in silage ensiled for 120 days. Inoculated silages (LB and Combo) had similar RA of transcripts related to *Firmicutes* (average of 97.05%) and *Proteobacteria* (average of 1.88%). In addition, LB and Combo had a greater *Firmicutes* RA (97.05 vs. 56.81%, *p* < 0.05) and lower *Proteobacteria* RA (1.88 vs. 28.93%, *p* < 0.05) than Control. Concerning all the genera found at a RA of more than 0.5% of the total population, no differences were observed between LB and Combo. However, inoculated silages had a lower (*p* < 0.05) RA of transcripts related to *Leuconostoc* (0.02 vs. 3.27%) and to *Escherichia* (0.49 vs. 7.87%) and greater (*p* < 0.05) RA of transcripts related to *Lactobacillus* (96.30 vs. 35.26%) than Control (taxonomic identification was made prior to the revised taxonomic proposition for new genera within *Lactobacillaceae*; Zheng et al., 2020). The number of LAB culturable in agar was measured at day 120 of ensiling. Inoculation affected the number of LAB (*p* = 0.001, SEM = 0.08), with LB (7.37 log_10_ cfu/g of fresh weight) and Combo (7.28 log_10_ cfu/g of fresh weight) having more LAB (*p* < 0.05) than Control (5.81 log_10_ cfu/g of fresh weight).

**Figure 5:**
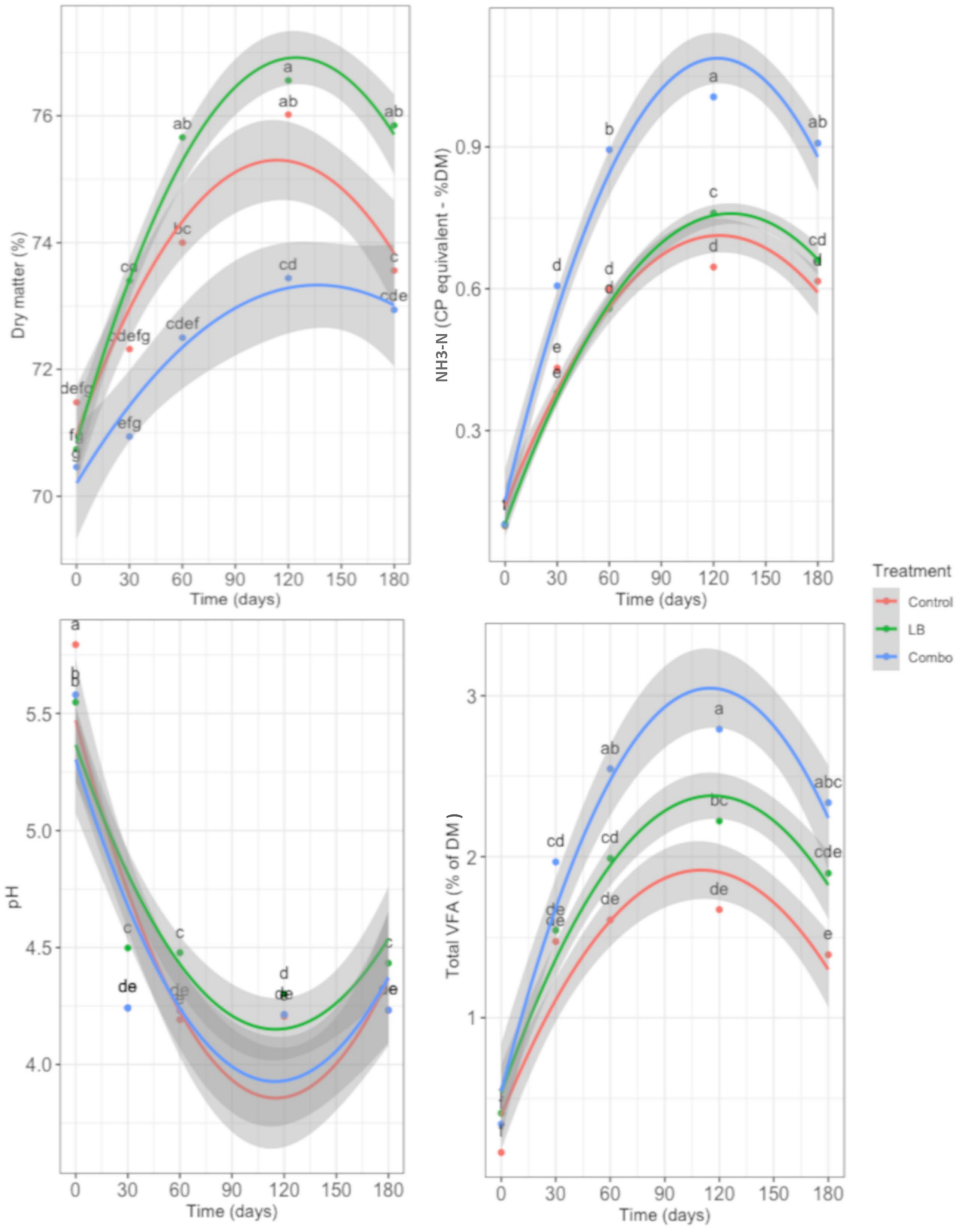
Changes in dry matter, NH_3_-N, pH, and total volatile fatty acids (VFA) over time (0, 30, 60, 120, and 180 d) in uninoculated (Control) silage or silage inoculated with *L. buchneri* NCIMB 40788 at 400,000 CFU/g of fresh weight (LB) or with *L. buchneri* NCIMB 40788 plus *L. hilgardii* CNCM-I-4785 at 200,000 CFU/g of fresh weight each (Combo). Interactions (*p* < 0.01) between treatment and time were observed for all items. Data points with different letters indicate a significant difference (*p* < 0.05).

**Figure 6:**
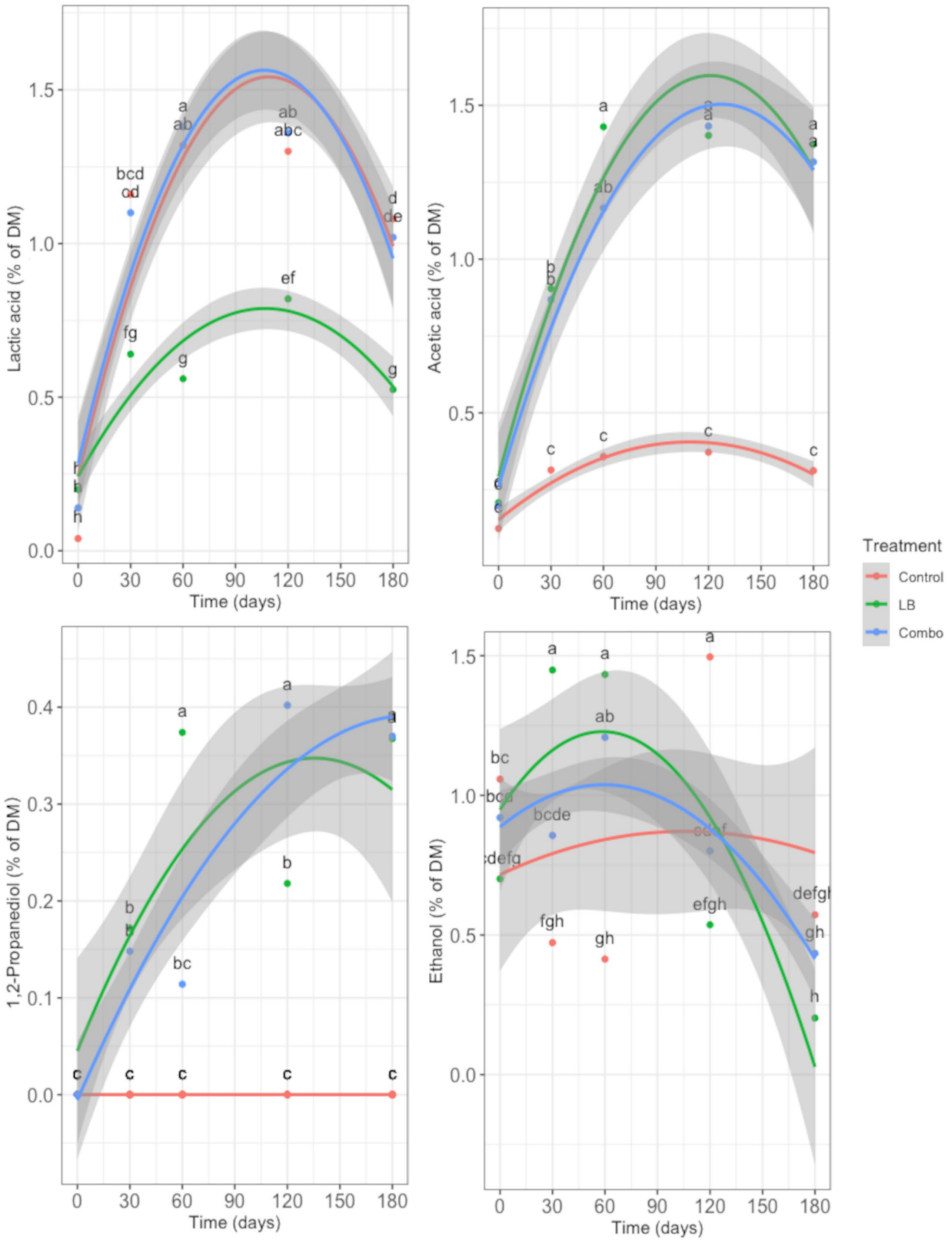
Changes in the concentration of lactic acid, acetic acid, 1,2-propanediol, and ethanol over time (0, 30, 60, 120, and 180 d) in uninoculated (Control) silage or silage inoculated with *L. buchneri* NCIMB 40788 at 400,000 CFU/g of fresh weight (LB) or with *L. buchneri* NCIMB 40788 plus *L. hilgardii* CNCM-I-4785 at 200,000 CFU/g of fresh weight each (Combo). Interactions (*p* < 0.001) between treatment and time were observed for all items. Data points with different letters indicate a significant difference (*p* < 0.05).

The changes in the fermentation profile during ensiling caused by inoculation are shown in Figures 5, 6. Interaction between treatment and time was observed for DM content (*p* = 0.003), NH_3_-N content (*p* < 0.001), pH (*p* < 0.001), and concentration of total VFA (*p* = 0.001; Figure 5). Regardless of the treatment, all fresh forage had similar DM content at day 0. The DM content of the silage increased from day 0 to day 60 (*p* < 0.05) regardless of the treatment, but the increase was greater (*p* < 0.05) in LB than in Combo and Control. Dry matter content remained stable in LB and Combo but decreased (*p* < 0.05) in Control from day 120 to day 180. The Combo treatment had a lower (*p* < 0.05) DM content than the LB treatment from day 30 and then the Control treatment at days 30, 60, and 120. The concentration of NH_3_-N was similar in all treatments at day 0 and increased (*p* < 0.05) up to day 60 in Control and up to day 120 in LB and Combo. The concentration of NH_3_-N was in the same range as that reported in published results (Gomes et al., 2020). The increase in the concentration of NH_3_-N was greater (*p* < 0.05) in Combo than in Control and LB. The concentration of NH_3_-N remained stable from day 120 to day 180 in all treatments. At day 0, the pH of Control was higher (*p* < 0.05) than the pH of LB and Combo. After 30 days of ensiling, the pH declined noticeably (*p* < 0.05) in all treatments, but the drop was less intense in LB than in Control and Combo. At all stages of the ensiling period, LB had a higher pH (*p* < 0.05) than Control and Combo, except at day 120, when the pH of LB and Combo were similar. The total VFA concentration increased (*p* < 0.05) from day 0 to day 30 and was similar among treatments at both time points. From day 30 to day 60, the total concentration of VFA continued to increase (*p* < 0.05) in Combo, whereas in Control and LB, the levels remained similar to those measured at day 30. From day 60 on, the total VFA concentration remained stable in all three treatments. The total concentration of VFA in Combo remained greater (*p* < 0.05) than that in Control and LB from day 60 to 120, but at day 180, the concentration in Combo was only higher than that in Control, and was similar to that in LB.

An interaction between treatment and time was observed in the concentrations of lactic acid, acetic acid, 1,2-propanediol, and ethanol (*p* < 0.001; Figure 6). At day 0, the concentration of lactic acid was similar in all three treatments. However, after ensiling, only Control and Combo had similar lactic acid concentrations, which were higher (*p* < 0.05) than that of LB at all time points. At day 0, the concentration of acetic acid was similar in all three treatments, but while it remained low in Control throughout the ensiling period, in LB and Combo, it increased (*p* < 0.05) up to day 60 and from then on, remained stable. Glycol 1,2-propanediol was not detected in Control at any time point evaluated, whereas in LB and Combo, 1,2-propanediol was not detectable at day 0 but its concentration subsequently increased throughout the ensiling period. The concentration of 1,2-propanediol increased (*p* < 0.05) faster in LB than in Combo. The concentration of ethanol was similar in all treatments at day 0 and it had an irregular pattern during ensiling in Control. In Combo, the concentration of ethanol remained stable from day 0 to 60, and then decreased (*p* < 0.05) until 180 d, whereas in LB, it increased (*p* < 0.05) from day 0 to 30, remained stable from day 30 to 60, and then decreased (*p* < 0.05). During the early stages of ensiling, the concentration of ethanol increased faster in LB than in Combo but was similar in the two treatments from day 60 on.

**Figure 7:**
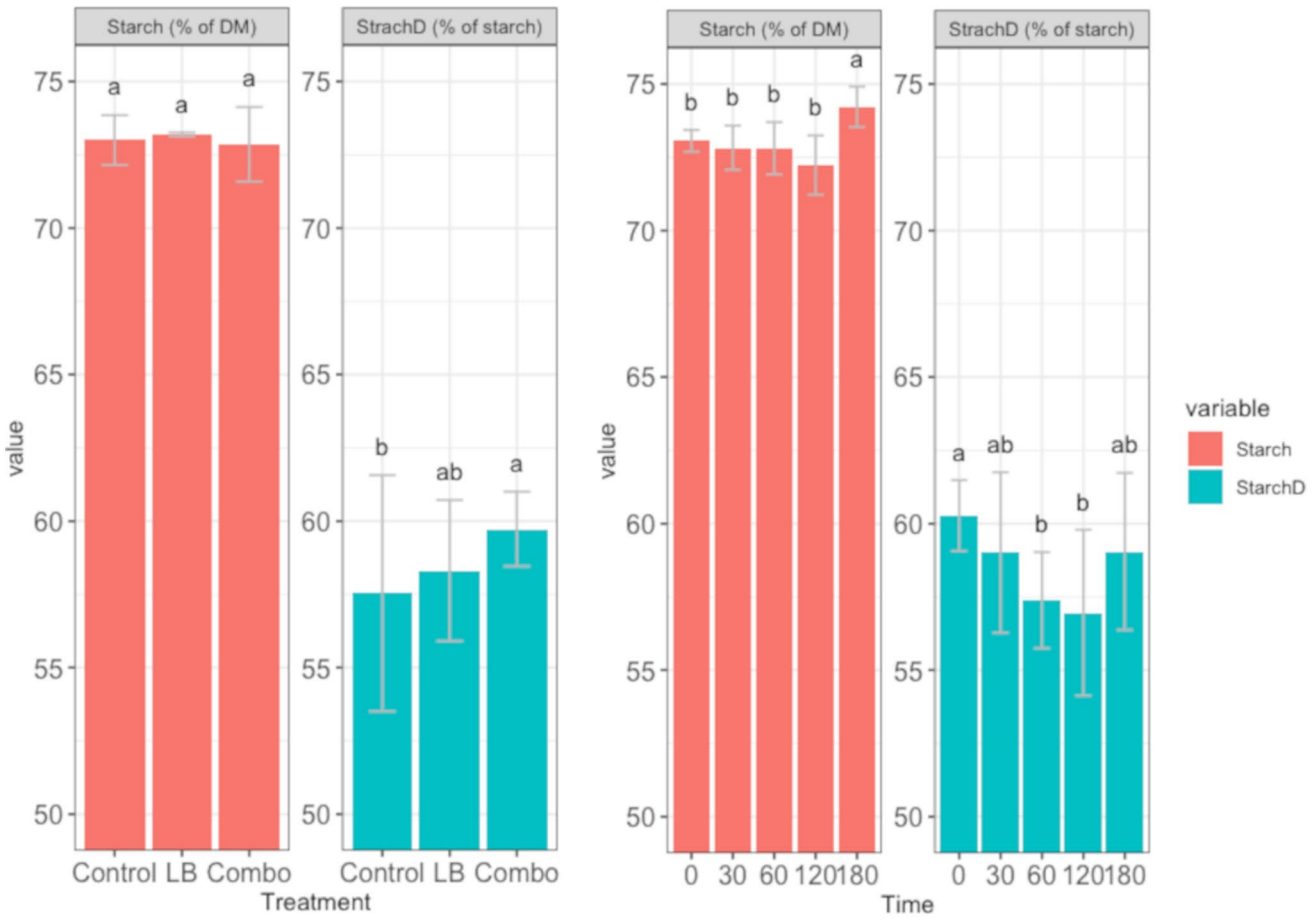
Effects of treatment (Control, uninoculated silage; LB, silage inoculated with *L. buchneri* NCIMB 40788 at 400,000 CFU/g of fresh weight; Combo, silage inoculated with *L. buchneri* NCIMB 40788 plus *L. hilgardii* CNCM-I-4785 at 200,000 CFU/g of fresh weight each) and time (0, 30, 60, 120, and 180 d) on starch content and starch digestibility (StarchD). Bars with different letters indicate a significant difference (*p* < 0.05). Starch concentration: treatment effect *p* = 0.230 and SEM = 0.16 and time effect *p* < 0.001 and SEM = 0.21). Starch digestibility: treatment effect *p* = 0.002 and SEM = 0.45 and effect of time *p* < 0.001 and SEM = 0.59.

The level of starchD measured was in the range obtained in high-moisture corn with the same moisture level (Ferraretto et al., 2014; Gallo et al., 2022). There was no interaction between treatment and time in the concentration of starch (*p* = 0.775) or starchD (*p* = 0.729). However, the concentration of starch was affected by time (*p* < 0.001) but not by treatment (*p* = 0.230), and starchD was affected by both time (*p* < 0.001) and treatment (*p* = 0.002; Figure 7). StarchD was higher (*p* < 0.05) in Combo than in Control but did not differ between LB and the other two treatments. The concentration of starch remained stable from day 0 to 120, but at day 180, it was higher (*p* < 0.05) than at the other time points. StarchD did not change from day 30 to 180 but was higher in fresh forage than in silages ensiled for 60 and 120 days.

**Figure 8:**
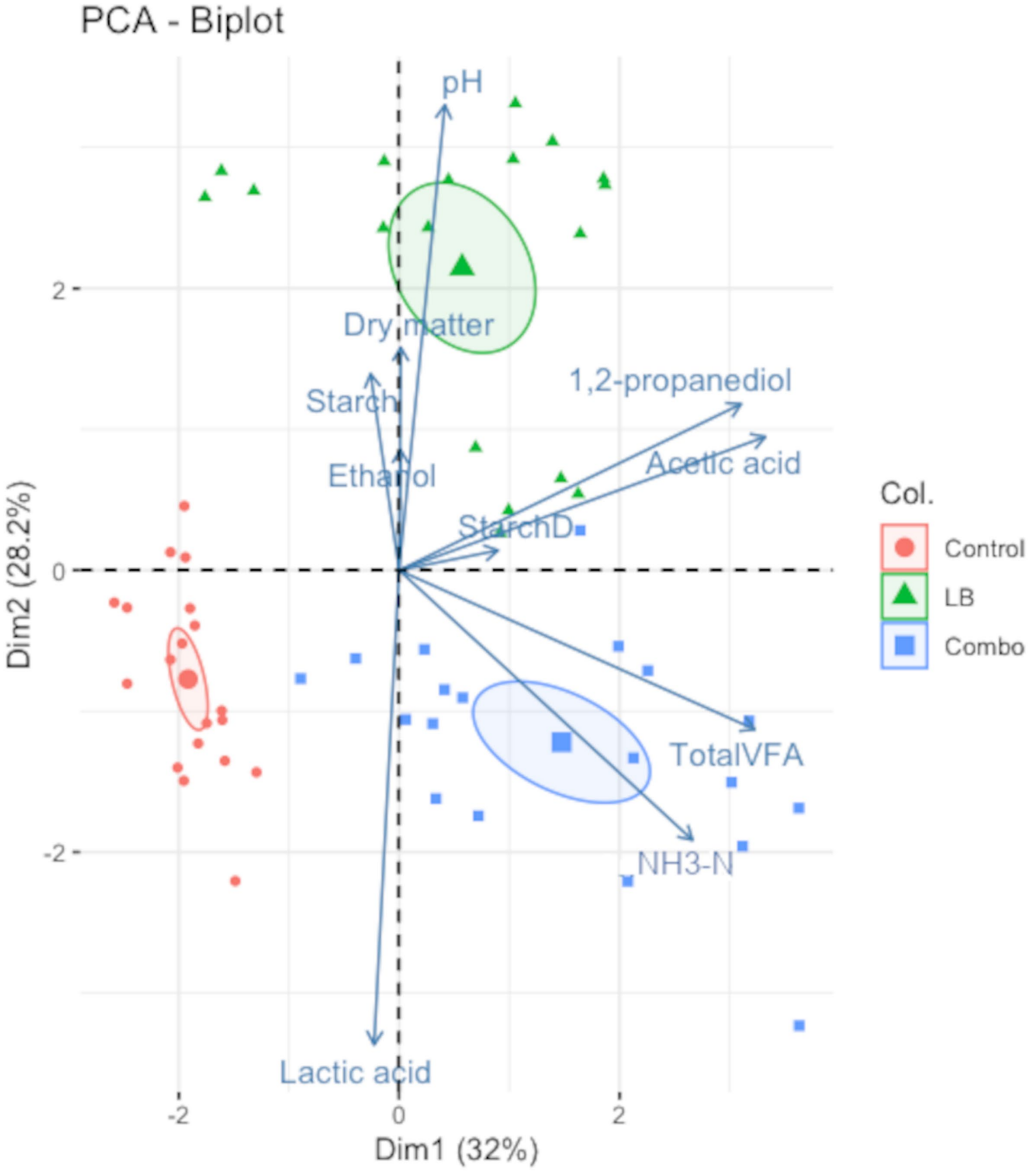
Principal component analysis (PCA) biplot showing the relationship between silage samples (dots) and chemical variables (arrows). Dimensions 1 and 2 were significant (*p* < 0.05) and accounted for 60.2% of the total variance. The variables NH_3_-N, total VFA, acetic acid, and 1,2-propanediol had significant (*p* < 0.05) loadings on dimension 1 while the variables NH_3_-N, pH, and lactic acid had significant loadings on dimension 2. Treatments consisted of uninoculated (Control) silage or silage inoculated with *L. buchneri* NCIMB 40788 at 400,000 CFU/g of fresh weight (LB) or with *L. buchneri* NCIMB 40788 plus *L. hilgardii* CNCM-I-4785 at 200,000 CFU/g of fresh weight each (Combo) ensiled for 30, 60, 120, and 180 days.

Figure 8 shows the PCA biplot of the relationship between silage variables at days 30, 60, 120, and 180 and the inoculation treatment. The first three dimensions were significant (*p* < 0.05). Dimensions 1 and 2 explained 60.2% of the total variance. The variables acetic acid, 1,2-propanediol, total VFA, and NH_3_-N significantly (*p* < 0.05) influenced the first dimension, while lactic acid, pH, and NH_3_-N significantly (*p* < 0.05) influenced dimension 2. Three distinct clusters formed, one for each treatment. The Control and Combo treatments were similar in that both were correlated with high lactic acid concentration and low pH. However, Combo was also correlated with high concentrations of total VFA, NH_3_-N, acetic acid, and 1,2-propanediol, whereas Control was not. Like Combo, LB was correlated with high concentrations of 1,2-propanediol and acetic acid, but, unlike Combo, LB was correlated with a high pH.

**Figure 9:**
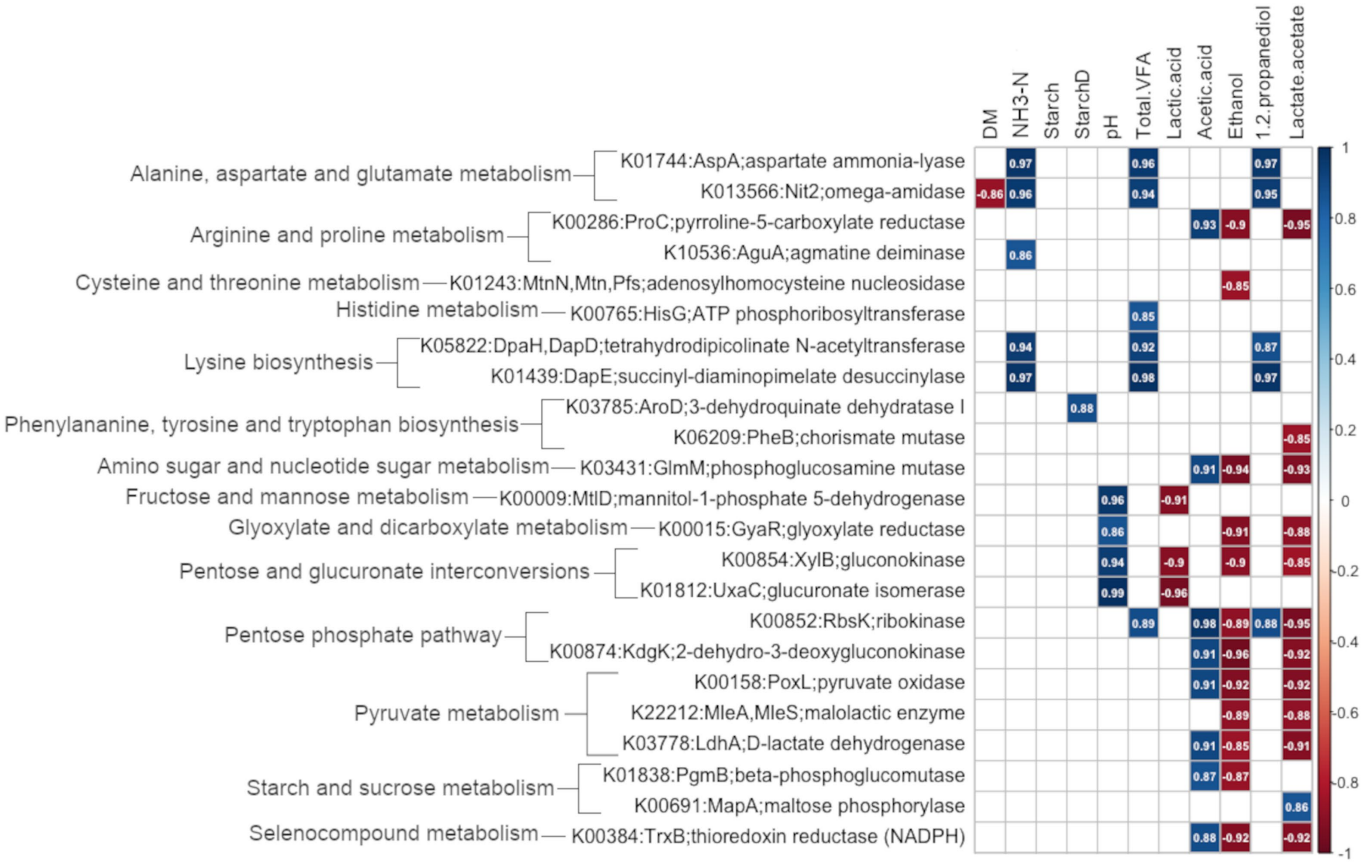
Pearson’s correlation matrix of the expression of genes of interest and chemical variables in silage ensiled for 120 days. The analysis included all treatments: Control (uninoculated), LB (inoculated with *L. buchneri* NCIMB 40788 at 400,000 CFU/g of fresh weight), and Combo (inoculated with *L. buchneri* NCIMB 40788 plus *L. hilgardii* CNCM-I-4785 at 200,000 CFU/g of fresh weigh each). Only genes that were significantly (*P*adj < 0.05) correlated with one or more chemical variables are shown. The colored squares indicate significant (*P*adj < 0.05) correlations and the numbers in the boxes indicate the correlation coefficient, *r*. The color red represents negative correlations and the color blue, positive correlations.

A correlation analysis was performed between the expression of the 69 genes of interest associated with amino acid and carbohydrate metabolism and the physicochemical variables of uninoculated and inoculated silage ensiled for 120 days, and the results are plotted in Figure 9. Of the 69 genes, 23 presented significant correlations (*P*adj < 0.05) with one or more physicochemical variables. Overall, 57 correlations were significant. No correlations were found between the genes analyzed and starch concentration, but all the other variables were correlated with at least one gene.

Dry matter content was negatively correlated with the expression of *nit2*, a regulatory gene involved in alanine, aspartate, and glutamate metabolism. Ammonia-N was positively correlated with the expression of several genes associated with amino acid metabolism, including *aspA* and *nit2* (alanine, aspartate, and glutamate metabolism) and *aguA*, *dapH*/*dapD*, and *dapE* (arginine and proline metabolism). StarchD was positively correlated with the gene *aroD*, associated with phenylalanine, tyrosine, and tryptophan biosynthesis. The genes *mtlD* (fructose and mannose metabolism), *xylB* (pentose and glucuronate interconversions), and *uxaC* (pentose and glucuronate interconversions) were positively correlated with pH and negatively correlated with lactic acid. Both total VFA and 1,2-propanediol were positively correlated with *rbsK*, which is associated with the pentose phosphate pathway, *dapE* and *dapH*/*dapD*, which are associated with lysine biosynthesis, and *aspA* and *nit2*, associated with alanine, aspartate, and glutamate metabolism. Total VFA was also positively correlated with *hisG* (histidine metabolism). Several genes were negatively correlated with the concentrations of ethanol and with the lactate-to-acetate ratio (except for *mapA*, which was positively correlated with the lactate-to-acetate ratio). Most of the genes that negatively correlated with ethanol concentration and with the lactate-to-acetate ratio were positively correlated with acetic acid: genes *poxL* and *ldhA* (pyruvate metabolism), *rbsK* and *kdgK* (pentose phosphate pathway), *glmM* (amino sugar and nucleotide sugar metabolism), *proC* (arginine and proline metabolism), and *trxB* (selenocompound metabolism)The gene *pgmB* associated with starch and sucrose metabolism was positively correlated with acetic acid but not significantly correlated with ethanol or the lactate-to-acetate ratio. Other genes involved in carbohydrate metabolism were negatively correlated with ethanol and with the lactate-to-acetate ratio (*mleA*/*mleS*, pyruvate metabolism; *gyaR*, glyoxylate and dicarboxylate metabolism; *xylB*, pentose and glucuronate interconversions) but not significantly correlated with acetic acid. In addition, ethanol was negatively correlated with the gene *mtnN* associated with the metabolism of the amino acids cysteine and methionine, and the lactate-to-acetate ratio was negatively correlated with the gene *pheB* associated with biosynthesis of the amino acids phenylalanine, tyrosine, and tryptophan, and positively correlated with the gene *mapA* associated with starch and sucrose metabolism.

## 4. Discussion

This study aimed to deepen our understanding of the interactions between *L. buchneri* and *L. hilgardii* when used as co-inoculants in high-moisture corn ensiling. The Combo treatment containing both strains was compared to the Control and LB treatments, the latter being used as a positive control. To investigate differences between the treatments, we analyzed the composition of the bacterial community, gene expression, and the fermentation profile of the silage. The bacterial community composition and gene expression were evaluated at day 120 of ensiling, while the biochemical composition and fermentation profile was evaluated at days 0, 30, 60, 120, and 180.

### 4.1. Differences between inoculated and uninoculated silages

Inoculation enhanced the development of the LAB population, as inoculated silages had more LAB culturable on agar plates and a greater RA of transcripts related to *Lactobacillus* than Control at day 120. Similarly, Ferrero et al. (2023) also observed an increased RA of *Lactobacillus* in HMC treated with *L. buchneri* and *L. hilgardii* in comparison to untreated HMC after 250 d of ensiling. The dominance of *Lactobacillu*s in inoculated silages reduced the RA of transcripts linked to *Leuconostoc* and *Escherichia.* In line with our results, *Escherichia* has already been shown to be inhibited by low pH and by the pH-independent antibacterial activity of certain *Lactobacillus* species, including *L. buchneri* (Blasel et al., 2006; Ferraretto et al., 2014). Establishing a robust LAB population and controlling the development of *Escherichia* in HMC is desirable, as this genus comprises strains, such as *Escherichia coli* O157:H7, that are detrimental to the nutritive value and hygienic-sanitary quality of silage (Queiroz et al., 2018).

In addition to its effects on the composition of the bacterial community, inoculation with LAB strains also affected gene expression at day 120 of ensiling. Inoculation intensified transcription rates, as indicated by the greater contig richness in Combo and LB compared to Control. Moreover, inoculation affected contig composition, as inoculated silage clustered separately from uninoculated silage in the PCoA plot.

According to Guo et al. (2023) the main metabolic pathways associated to silage fermentation are related to the metabolism of amino acids, carbohydrates, nucleotides, and energy. We identified 10 genes involved in carbohydrate metabolism and six involved in amino acid metabolism that were differently expressed in Combo and LB compared to in Control at day 120 of ensiling Interestingly, all these genes were upregulated in inoculated silages. The upregulation of genes related to carbohydrate metabolism suggests that inoculation enhanced fermentation in HMC. Even though it is not possible to confirm if the upregulation of those genes is directly related to the activity of the inoculated strains or of other microorganisms that could have been stimulated by inoculation some hypotheses can be raised. In line with our findings, Bai et al. (2022) observed that genes associated with carbohydrate metabolism were upregulated in whole-plant corn silage inoculated with *L. buchneri* earlier during ensiling than in uninoculated silage (after 3 days vs. 7 days of ensiling when stored at 20°C, and after 1 day vs. 3 days when stored at 30°C). *L. buchneri* is incapable of fermenting carbohydrates via glycolysis and lacks genes coding for transaldolase and transketolase that are required for the pentose phosphate (PP) pathway (Heinl and Grabherr, 2017). Both *L. buchneri* and *L. hilgardii* are consequently classified as obligate heterofermentative LAB. The main carbohydrate fermentation route of these two LAB species is the pentose phosphoketolase (PPK) pathway, which produces equimolar amounts of CO_2_, lactate, and ethanol or acetate (Spector, 2009). The first steps in the PPK pathway are shared with the PP pathway (Spector, 2009) so that upregulation of genes involved in the PP pathway (*gntK*/*idnK*, *rbsK*, and *kdgK*) and pentose and glucuronate interconversions (*uxaC*) in inoculated silages compared to in Control were considered likely. The *kgdK* gene codes for a kinase that catalyzes the phosphorylation of 2-dehydro-3-deoxy-D-gluconate into 2-dehydro-3-deoxy-D-gluconate-6P, which can subsequently be converted into D-gluconate-6P and the *gntK*/*idnK* gene codes for a gluconokinase that catalyzes the phosphorylation of D-gluconate into D-gluconate-6P (Bausch et al., 1998; Martis et al., 2021). The gene *rbsK* codes for the enzyme ribokinase, which phosphorylates ribose to ribose 5-P (Park and Gupta, 2008). The products of the reactions previously described, D-gluconate-6-P and ribose 5-P, are precursors of ribulose-5-P, an intermediate in the non-oxidative branch of the PPK pathway (Spector, 2009). According to the KEGG database, the pentose and glucuronate interconversion pathway leads to the production of intermediates of the PP pathway and of intermediates of the PPK pathway. Therefore, upregulation of the gene *uxaC* involved in this pathway was also expected. Stimulation of the PPK pathway in the inoculated silages resulted in a higher acetic acid content than Control. In addition, there was a negative correlation between the lactate-to-acetate ratio and the genes *rbsK* and *kdgK*. A decrease in the lactate-to-acetate ratio indicates a shift toward obligate heterofermentative fermentation (Kung Jr et al., 2018).

The genes *ldhA* and *mleA*/*mleS*, associated with pyruvate metabolism, were upregulated in inoculated silages. The gene *ldhA* has been previously detected in *L. buchneri* (Okoye et al., 2022) and it codes for D-lactate dehydrogenase that catalyzes the reduction of pyruvate to lactate, the main step in the production of D-lactate in LAB in anaerobic conditions (Jia et al., 2018). The gene *mleA*/*mleS* codes for the malolactic enzyme that drives the fermentation of malate into lactate, the main fate of malate during the ensiling process (Rooke and Hatfield, 2003). Even though inoculation appeared to have stimulated lactate production, inoculated silages did not contain a higher concentration of lactate than Control. The conversion of lactate into acetate could explain why lactate did not over-accumulate in the inoculated silage. This hypothesis is corroborated by the upregulation of the *poxL* gene in inoculated silage. This gene codes for the enzyme pyruvate oxidase, which is involved in the aerobic accumulation of acetate in LAB (Guo et al., 2017). In addition, Heinl and Grabherr (2017) proposed a pathway in which the genes pox2 and pox3 (pyruvate oxidases) influenced the conversion of pyruvate into acetate in *L. buchneri* CD034 under aerobic conditions. In line with this proposal, we observed that the gene *poxL* was positively correlated with acetic acid and negatively correlated with ethanol. Upregulation of the gene *poxL* presumes that some oxygen was present in the silo bag, which is not surprising since the silo plastic is subject to degradation over long periods of time and oxygen can slowly permeate the plastic, both facts corresponding to the 120-day period that preceded sampling in our case.

The gene *gyaR* codes for the enzyme glyoxylate reductase that acts on the glyoxylate cycle. In bacteria, the glyoxylate cycle is used to enable growth by synthesizing carbohydrates from two carbon compounds (acetate and ethanol) when simple sugars, such as glucose or fructose, are not available (Kornberg, 1966). In the present study, the gene *gyaR* was positively correlated with pH and negatively correlated with the lactate-to-acetate ratio and was upregulated in inoculated silages. The upregulation of this gene in inoculated silages could be caused by the reduced availability of simple sugars presumably due to the development of *Lactobacillus* or by the increased availability of acetate (substrate for the glyoxylate cycle) in those silages compared to in Control. The gene *glmM*, associated with amino sugar and nucleotide sugar metabolism, was also upregulated in inoculated silages. This gene codes for the enzyme phosphoglucosamine mutase that catalyzes the interconversion of glucosamine-6-P and glucosamine-1-P and is essential for cell wall development and microbial growth (Barreteau et al., 2008). The gene *pgmB* was another gene associated with the synthesis of cell wall polysaccharides that was upregulated in inoculated silages. This gene codes for the enzyme β-phosphoglucomutase that catalyzes the conversion of β-D-glucose-1-P to β-D-glucose-6-P (Qian et al., 1994). The upregulation of the genes *glmM* and *pgmB* in inoculated silages could reflect an increased microbial development.

Inoculation also affected the expression of some genes involved in amino acid metabolism. All six genes that were differently expressed in LB and Combo compared to Control were upregulated in the inoculated silages ensiled for 120 days. In addition, according to our search of the KEGG database, all these genes have already been identified in both *L. buchneri* and *L. hilgardii*, except for the gene *thrB1*, which was not found in *L. hilgardii*. Some of the differently expressed genes were associated with microbial growth and could be associated with the development of the inoculants in the silage. One such gene was *dapE*, which codes for the N-succinyl-L, L-diaminopimelate desuccinylase, an enzyme that catalyzes a reaction in the final steps of lysine biosynthesis (Usha et al., 2016). Lysine is needed for protein synthesis and the construction of the peptidoglycan layer of the cell walls in Gram-positive bacteria, such as LAB (Gillner et al., 2013). Another upregulated gene was *thrB1*. This gene codes for homoserine kinase, which is involved in an important reaction in the biosynthesis of threonine and exopolysaccharide production (Zhou et al., 2000). Lactic acid bacteria produce exopolysaccharides as a protective mechanism against environmental stressors, such as pH, antibiotics, and temperature (Nguyen et al., 2020). In addition, the gene *aspA*, which has been previously identified in *L. buchneri* (Okoye et al., 2022) and codes for the enzyme aspartase, was also upregulated in inoculated silage. Aspartase catalyzes the interconversion of aspartate to fumarate and ammonia and. The fumarate produced in this reaction can be reduced to succinate by some LAB, thereby enabling regeneration of the intracellular NAD+ pool, which can be used to boost the use of sugar (Fernández and Zúñiga, 2006; Okoye et al., 2022). Some of the amino acid metabolism genes upregulated in inoculated silage were associated with stress tolerance, such as osmotic, aerobic, and pH stress. For example, we observed upregulation of the gene *proC*, which codes for pyroline-5-carboxylate reductase, an enzyme that catalyzes the final reaction of proline biosynthesis (Newsholme et al., 2011). This gene might have been upregulated because proline can act as an osmotic protectant in some LAB, as they accumulate this amino acid in response to osmotic stress (Baliarda et al., 2003). Even though HMC contains more moisture than dry corn, the water availability in HMC is nevertheless low and its osmotic stress is severe for most microorganisms. The gene *trxB* was also upregulated in inoculated silage. This gene is a common antioxidant-related gene present in the genome of *Lactobacillus* that codes for thioredoxin reductase, which is involved in the thioredoxin system as a key pathway of redox regulation and in protecting cells against oxygen in anaerobes (Zeller and Klug, 2006; Kang et al., 2023). Therefore, the upregulation of *trxB* is in line with the upregulation of *poxL*, which, as previously mentioned, was indicative of the presence of oxygen in the silo. The gene *aguA* that codes for agmatine deiminase was also upregulated in inoculated silages. Agmatine deiminase catalyzes the interconversion of agmatine and hydroxide peroxide to N-carbamoylputrescine and ammonia (Janowitz et al., 2003). Accordingly, the *aguA* gene was positively correlated with NH_3_-N. Agmatine deamination is hypothesized to be involved with intracellular pH-homeostasis in a similar way to the arginine deaminizing pathway, and both those pathways have been reported to be present in *L. buchneri* (Johanningsmeier and McFeeters, 2015; Heinl and Grabherr, 2017).

Several genes associated with quorum-sensing were upregulated in LB and Combo compared to Control at day 120 of ensiling, indicating that communication mechanisms were more active in inoculated silages. For example, the gene that codes for a Lon-like protein, an adhesin involved in intercellular adhesion (Wang et al., 2021), was upregulated in inoculated silages. In addition, genes associated with the recognition and internalization of environmental signals were also upregulated in inoculated silages. In general, Gram-positive bacteria, such as lactobacilli, use communicating peptides (autoinducing peptides) for quorum sensing, whereas, in Gram-negative bacteria, quorum sensing is usually mediated by N-acyl homoserine lactones (Kareb and Aïder, 2020). For this reason, upregulation of genes associated with Gram-positive bacteria quorum sensing in inoculated silages was expected, as the inoculants comprised Gram-positive bacteria and likely helped stimulate the whole *Lactobacillus* population, as evidenced by its dominant RA. The gene *oppD* was upregulated in inoculated silages. In Gram-positive bacteria, autoinducing peptides are internalized through oligopeptide transport systems (Opp), five-component ABC transporters (Waters and Bassler, 2005). The *oppD* gene codes for the oligopeptide transport system protein OppD that is bound to the membrane and provides energy for oligopeptide translocation (Gardan et al., 2009). In inoculated silages, we also observed upregulation of a gene coding for ABC peptide/nickel transport system substrate-binding proteins, and of the gene *yidC*, which codes for the YidC/Oxa1 family membrane protein insertase. The gene *yidC* has been associated with EPS composition and biofilm formation and with conditions characterized by low pH and lack of nutrients (Guo et al., 2018; Palmer et al., 2019). Unexpectedly, even though the protein YidC/Oxa1 is associated with the SecYEG translocon, the gene *secY,* which codes for the preprotein translocase subunit SecY, was downregulated in inoculated silages compared to Control. Since the LAB active in Control could also form biofilm, this observation does not explain the lack of activity required for these functions. The *lacD* gene was another gene upregulated in inoculated silages. This gene codes for a key enzyme in the tagatose-6-phosphate pathway, part of lactose and galactose metabolism. The gene *lacD* has been shown to be required for the colonization of certain species of Gram-positive bacteria and to be involved in gene expression regulation (Loughman and Caparon, 2006; Paixão et al., 2015). In addition, a link between the LuxS/AI-2 quorum-sensing system, involved in biofilm formation, and the tagatose-6-phosphate pathway genes *lacA*, *lacB*, *lacC*, *lacD*, and *lacG-2* has recently been proposed (Yadav et al., 2018).

### 4.2. Differences between combo and LB silages

At day 120 of ensiling, transcription rates were higher in Combo than in LB silage, as evidenced by the greater contig richness in Combo. However, contig diversity and the bacterial community were similar in the two treatments, with no differences in the RA of *Lactobacillus*. Several genes associated with carbohydrate metabolism, amino acid metabolism, and quorum sensing were differentially expressed in Combo and LB at day 120 of ensiling.

The main difference we observed between the fermentation of Combo and LB silage was that, whereas LB fermentation occurred as expected for an inoculant containing *L. buchneri*, Combo behaved unexpectedly for an inoculant containing obligate heterofermentative LAB. When silage is inoculated with *L. buchneri*, a decrease in lactic acid concentration is expected, as it is partially converted into acetic acid (Oude Elferink et al., 2001). This pattern was observed in LB, but in Combo, the lactic acid concentration remained high, i.e., at a level similar to that in Control, while high levels of acetic acid and 1,2-propanediol were still produced at a level similar to that in LB. In addition, compared to LB, Combo had a lower pH throughout ensiling due to its higher concentration of lactic acid. Similar findings were previously reported by Drouin et al. (2019) and Ferrero et al. (2019) when *L. buchneri* and *L. hilgardii* were tested alone and in combination in whole-plant corn silage. However, when analyzing these species in HMC, da Silva et al. (2021) did not obtain the same results as those of the present study. We consequently performed transcriptomics analysis to investigate the change in the fermentation profile when *L. hilgardii* was combined with *L. buchneri*.

Four genes associated with carbohydrate metabolism were differentially expressed in the two inoculation treatments, all four were downregulated in Combo compared to LB. As mentioned previously, the genes *gntK*/*idnK*, *uxaC*, and *uxaB* are involved in reactions that lead to the production of intermediates of the PPK pathway. Therefore, the downregulation of those genes in Combo may mean that the PPK pathway was less active in this silage than in LB at 120 d. A possible explanation is that the higher levels of lactate present in the Combo silage promoted a more intense product inhibition of the PPK pathway at 120 d in this silage compared to LB silage. In *L. buchneri,* low pH triggers the conversion of lactic acid into weaker acetic acid to protect the cell against high acidity (Oude Elferink et al., 2001). Oude Elferink et al. (2001) reported that when grown at pH 3.8, the conversion rate of lactate to acetate by *L. buchneri* was higher than when grown at pH 4.3. The higher accumulation of lactate and lower pH in Combo than in LB silage might indicate that compared to *L. buchneri*, *L. hilgardii* was more resistant to the acidic conditions and that lactate degradation might be induced at a lower pH in *L. hilgardii* than in *L. buchneri*. Such capability of the *L. hilgardii* and *L. buchneri* combination of reducing silage pH to a lower level than *L. buchneri* alone is advantageous as at a lower pH, a higher proportion of acetate is present in the undissociated form, which possesses antifungal capacity (Muck et al., 2018).

Another fact that needs taking into consideration is that we only analyzed the transcriptome at day 120 of ensiling, and consequently only dispose of a snapshot of what was occurring in the silo at that specific moment. In addition, *L. buchneri* and *L. hilgardii* were not necessarily active in the silo at the same time. Therefore, one species could have developed earlier than the other, which may have influenced gene expression when the sample was collected. In addition, Combo and LB may have stimulated the activity of different epiphytic silage microorganisms, which could also have affected gene expression at day 120 as well as the fermentation profile. For example, Drouin et al. (2019) evaluated the effects of *L. buchneri* and *L. hilgardii* alone or in combination in whole-plant corn silage and observed that inoculation using the combination resulted in silage with lower alpha diversity, while inoculation with *L. buchneri* alone resulted in more diverse LAB after 64 d of ensiling.

Still, concerning carbohydrate metabolism, the gene coding for the enzyme alpha, alpha-trehalose phosphorylase, associated with starch and sucrose metabolism, tended to be upregulated in Combo more than in LB. This enzyme catalyzes the degradation of trehalose to D-glucose and beta-D-glucose-1-P (Sun and You, 2021). Trehalose is a product of starch degradation (Yoshida et al., 1997) and for this reason, upregulation of a gene associated with trehalose metabolism in Combo might be associated with increased starchD. This observation would corroborate the fact that starchD was improved by Combo treatment but not by LB treatment when compared to Control. In addition, we observed that, compared to LB silage, Combo had a higher concentration of NH_3_-N and a higher expression of the genes *aspA* and *nit2*, which are usually associated with aspartate metabolism. These observations could be linked to improved degradation of the protein (zein)-starch matrix of the corn kernels and, consequently, increased starchD. The protein-starch matrix of the corn kernels has to be broken down to increase starchD, and the concentration of NH_3_-N in the silage may increase as a result of this process (Rooke and Hatfield, 2003; Cueva et al., 2023). In addition, corn proteins are rich in aspartate (Belitz et al., 2009) therefore, the upregulation of genes associated with aspartate catabolism could also indicate increased zein proteolysis. In another connection, aspartate is a precursor of lysine biosynthesis (Møller, 1974). The gene *dapE*, associated with lysine biosynthesis, was upregulated in Combo. As mentioned previously, lysine is an amino acid that is required for protein synthesis and construction of the peptidoglycan layer of the cell walls of Gram-positive bacteria (Gillner et al., 2013). Therefore, because corn is deficient in lysine, silage bacteria have to synthesize this amino acid to grow. Further illustrated by a more active crosstalk between bacteria in Combo, the greater ability in producing lysine can also contribute to explain the higher alpha diversity reported for Combo.

The gene *yajC* was the only gene associated with quorum sensing that was differentially expressed in LB and Combo. This gene codes for the preprotein translocase subunit YajC, which has been associated with ethanol resistance in *L. buchneri* (Liu et al., 2016) and was found to be upregulated in LB. However, when the samples were collected for transcriptomics analysis at day 120, the ethanol concentration in LB and Combo was similar, so we were unable to identify the reason for the upregulation of this gene in LB.

Transcriptomics analysis was useful as it helped clarify the causes of the differences in fermentation between Combo and LB. However, in the present study, we only evaluated gene expression at day 120 of ensiling. We believe that to better understand the shifts in microbial activity during ensiling, future research should investigate transcriptome dynamics throughout ensiling, especially in the early stages when most changes occur. In addition, in the future, transcriptomics analysis could be applied to deepen our knowledge of how different inoculants affect the activity and gene expression of epiphytic LAB.

Even though metatranscriptomics analysis was performed only at 120 d, it helped further our understanding of how an inoculant combining *L. buchneri* and *L. hilgardii* affected HMC ensiling. Overall, contig beta-diversity and the composition of the bacterial community did not differ between Combo and LB, but inoculation with Combo intensified transcription rates compared to LB. Several genes associated with amino acid degradation were upregulated in Combo, and a gene associated with starch and sucrose metabolism tended to be upregulated in Combo. In Combo, inoculation seemed to have intensified proteolysis compared to Control and LB, thereby increasing starchD compared to Control, whereas LB has similar starchD content to Control. Genes associated with the PPK pathway were downregulated in Combo. Whereas Combo had similar concentrations of acetic acid and 1,2-propanediol to LB, it had a higher concentration of lactic acid and a lower pH.

## Data availability statement

The datasets presented in this study can be found in online repositories. The names of the repository/repositories and accession number(s) can be found at https://www.ncbi.nlm.nih.gov/, PRJNA880369.

## Author contributions

PD: Conceptualization, Data curation, Investigation, Methodology, Project administration, Writing – reviewing & editing. ÉS: Formal analysis, Visualization, Writing – original draft, Writing – reviewing & editing. JT: Data curation, Investigation, Writing – reviewing & editing. EC: Writing – reviewing & editing. EA: Writing – reviewing & editing. MC: Writing – reviewing & editing.

## Footnotes

^1^ sourceforge.net/projects/bbmap

^2^ http://bio-bwa.sourceforge.net

^3^ github.com/jtremblay/MicrobiomeUtils
